# RGL2 Drives the Metastatic Progression of Colorectal Cancer via Preventing the Protein Degradation of β-Catenin and KRAS

**DOI:** 10.3390/cancers13081763

**Published:** 2021-04-07

**Authors:** Meng-Shun Sun, Lan-Ting Yuan, Chia-Hao Kuei, Hui-Yu Lin, Yen-Lin Chen, Hui-Wen Chiu, Yuan-Feng Lin

**Affiliations:** 1 Department of Internal Medicine, Division of Gastroenterology, Yuan’s General Hospital, Kaohsiung 80249, Taiwan; dtmed655@yahoo.com.tw (M.-S.S.); emyuan@hotmail.com (L.-T.Y.); 2 Graduate Institute of Clinical Medicine, College of Medicine, Taipei Medical University, Taipei 11031, Taiwan; pplay1028@gmail.com (C.-H.K.); candycarol0227@gmail.com (H.-Y.L.); 3 Department of Urology, Division of Surgery, Cardinal Tien Hospital, Xindian District, New Taipei City 23148, Taiwan; 4 Department of Breast Surgery and General Surgery, Division of Surgery, Cardinal Tien Hospital, Xindian District, New Taipei City 23148, Taiwan; 5 Department of Pathology, Cardinal Tien Hospital, School of Medicine, College of Medicine, Fu-Jen Catholic University, New Taipei City 23148, Taiwan; anthonypatho@gmail.com; 6 Department of Medical Research, Shuang Ho Hospital, Taipei Medical University, New Taipei City 23561, Taiwan; 7 TMU Research Center of Urology and Kidney, Taipei Medical University, Taipei 11031, Taiwan; 8 Cell Physiology and Molecular Image Research Center, Wan Fang Hospital, Taipei Medical University, Taipei 11696, Taiwan

**Keywords:** colorectal cancer, metastasis, RGL2, Wnt/β-catenin, KRAS

## Abstract

**Simple Summary:**

Aberrant activation of the Wnt/β-catenin pathway due to APC (adenomatous polyposis coli) loss and Kirsten ras (KRAS) mutation is highly associated with malignant evolution, e.g., metastasis, of colorectal cancer (CRC). Ral guanine nucleotide dissociation stimulator-like (RGL) proteins, such as RGL2, regulate RAS activity via controlling the exchange between GTP and GDP. Although the cross-talk between β-catenin and KRAS has been reported to promote cancer metastasis, the functional role of RGL2 remains largely unknown. Here we show that RGL2 is significantly upregulated in primary tumors compared to normal tissues and serves as a poor prognostic marker in CRC patients. Cell-based and animal experiments further demonstrate that RGL2 acts as a driver to promote the metastatic progression of CRC, most likely via preventing the protein degradation of β-catenin and KRAS. Our findings not only unveil the oncogenic function of RGL2 but also provide a new strategy to combat metastatic CRC by targeting RGL2 activity.

**Abstract:**

Colorectal cancer (CRC) is one of the most common cancers and results in high mortality worldwide, owing to cancer progression, i.e., metastasis. However, the molecular mechanism underlying the metastatic evolution of CRC remains largely unknown. Here, we find that the upregulation of Ral Guanine Nucleotide Dissociation Stimulator Like 2 (RGL2) is commonly detected in primary tumors compared normal tissues and is significantly associated with a poorer prognosis in CRC patients. Moreover, RGL2 expression appeared to positively correlate with the metastatic potentials of CRC cells. Whereas RGL2 knockdown dramatically suppresses the metastatic potentials of CRC cells in vitro and in vivo, RGL2 overexpression in the poorly metastatic CRC cells and reconstitution in the RGL2-silenced CRC cells enhanced and rescued the cellular metastatic ability, respectively. Computational simulation using Gene Set Enrichment Analysis program and cell-based assays demonstrated that RGL2 expression causally associated with the activity of Wnt/β-catenin signaling axis and Kirsten ras (KRAS)S, as well as the progression of epithelial-mesenchymal transition (EMT) in the detected CRC cells. Importantly, RGL2 upregulation was capable of preventing the protein degradation of β-catenin and KRAS in CRC cells. These findings suggest that RGL2 acts as a driver to promote the metastatic progression of CRC and also serves as a poor prognostic biomarker in CRC patients.

## 1. Introduction

Colorectal cancer (CRC) is the third most common cancer and the second leading cause of cancer-related death in Western countries, with more than 600,000 deaths worldwide each year [1]. Death resulting from CRC is associated with the disease stage, a more advanced grade, and the presence of obstruction [2]. Currently, two groups of CRC have been identified by molecular pathological characteristics [3,4]. The first group consists of approximately 16% of hypermutated tumors harboring microsatellite instability (MSI) due to defective mismatch repair (MMR). The second group belongs to nonhypermutated tumors (~84%) that are microsatellite stable (MSS) cancers with a high frequency of DNA somatic copy number alterations (SCNAs) and dysregulated Wnt pathway with frequent mutations in genes including adenomatous polyposis coli (APC) and Kirsten ras (KRAS). APC mutation is known to force the activation of β-catenin signaling cascades in CRC [5]. Several lines of evidence have shown that APC and KRAS mutations foster CRC progression, e.g., metastasis [6,7,8,9]. The interaction between β-catenin and mutant KRAS upon APC mutation was found to increase their protein stability and promote the metastatic progression of CRC [10]. Therefore, the simultaneous destabilization of β-catenin and mutant KRAS has been considered an effective anticancer strategy for CRC with APC mutations.

Ral guanine nucleotide dissociation stimulator-like (RGL) proteins consist of four subtypes, namely, RGL1, RGL2, RGL3, and RGL4. They are characterized as guanyl-nucleotide exchanging factors (GEFs), which regulate the exchange between GTP and GDP and are associated with the regulation of RAS activity [11]. A recent report showed that a decreased level of RGL4 is correlated with a poor prognosis and immune infiltration in lung adenocarcinoma [12]. In KRAS mutant pancreatic ductal adenocarcinoma (PDAC), an increased level of RGL2 was detected in tumors compared to normal tissues and was associated with PDAC metastatic capacity [13]. Nevertheless, the role of RGL2, as well as other RGLs, in KRAS-mediated CRC progression remains largely unknown.

Therefore, this study attempted to delineate the clinical relevance and oncogenic function of RGL2 in CRC. We found that RGL2 upregulation is extensively detected in CRC compared to normal tissues and significantly correlated with a poorer prognosis in CRC patients. Moreover, RGL2 knockdown was suppressed, but overexpression promoted the metastatic potential of CRC cells in vitro and in vivo by fostering the progression of epithelial-mesenchymal transition and the activity of β-catenin. Importantly, our results suggest that RGL2 upregulation is capable of preventing the protein degradation of β-catenin and KRAS and thereby stabilizing the β-catenin and KRAS signaling pathways in metastatic CRC. This study is the first to document the oncogenic role of RGL2 in promoting cancer progression.

## 2. Results

### 2.1. RGL2 Is Upregulated in Colorectal Cancer Compared to Normal Adjacent Tissues

**Figure 1:**
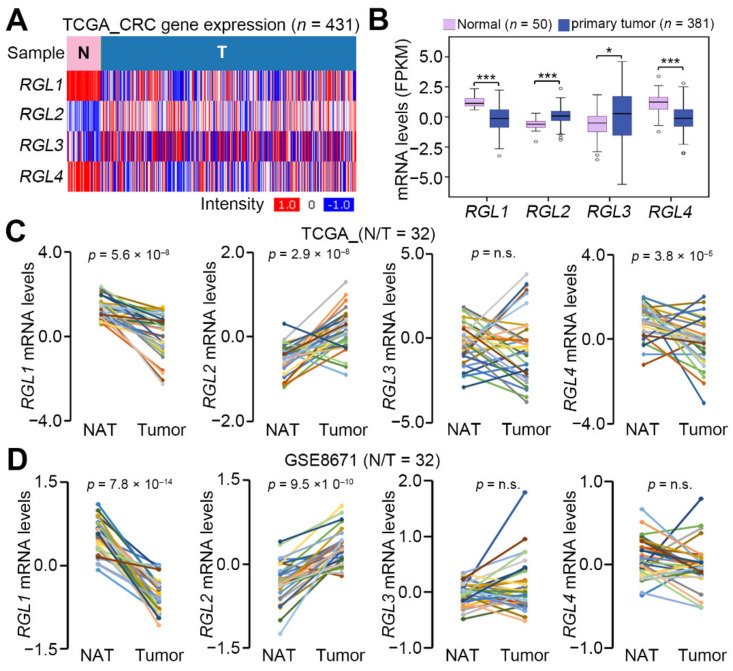
Ral Guanine Nucleotide Dissociation Stimulator Like 2 (RGL2) is upregulated in primary tumors compared to normal tissues derived from colorectal cancer patients. Heatmap (**A**) and boxplots (**B**) for the transcription profiling of RGL1, RGL2, RLG3, and RGL4 in the The Cancer Genome Atlas (TCGA) colorectal cancer database. The statistical significance was analyzed using a t-test. The symbols “*” and “***” represent *p* < 0.05 and *p* < 0.001, respectively. The mRNA levels of RGL1, RGL2, RLG3, and RGL4 in normal adjacent tissues (NATs) and primary tumors from the TCGA (**C**) and GSE8671 colorectal cancer patients (**D**). The statistical significance was evaluated by paired *t*-tests. The “n.s.” denotes not significant.

We first analyzed the transcriptional profiling of RGL subtypes RGL1, RGL2, RGL3, and RGL4 in The Cancer Genome Atlas (TCGA) colorectal cancer (CRC) database. The data showed that the mRNA levels of RGL2 and RGL3 in primary tumors were significantly (*p* < 0.001) higher than those in normal tissues, whereas RGL1 and RGL4 were downregulated in primary tumors compared to normal tissues in the TCGA CRC database (Figure 1A,B). The transcriptional profiling of RGL genes further demonstrated that the mRNA levels of RGL2, not RGL3, were significantly (*p* < 0.001) upregulated, but RGL1 and RGL4 were predominantly downregulated in primary tumors compared to normal adjacent colorectal tissues (Figure 1C). Similar results were validated in the paired normal adjacent tissues and primary tumors derived from the GSE8671 CRC dataset (Figure 1D). Based on these findings, we thereafter focused on investigating the clinical relevance and oncogenic role of RGL2 in CRC. 

### 2.2. RGL2 Upregulation Correlates with a Poorer Prognosis in CRC Patients

**Figure 2:**
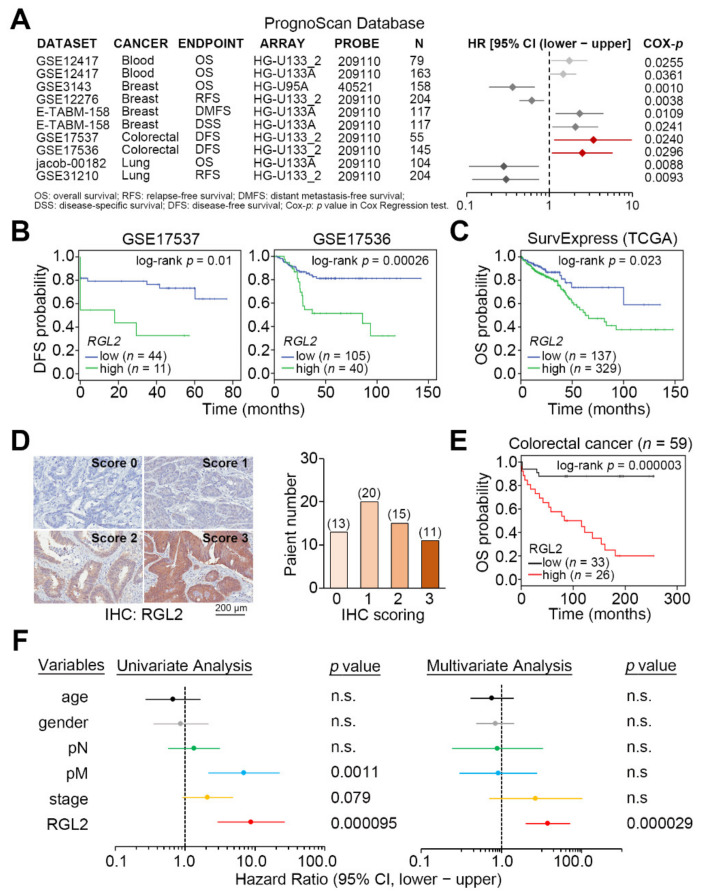
RGL2 upregulation refers to a poorer prognosis in colorectal cancer (CRC) patients. (**A**) Forest plot for the hazard ratio (HR) of RGL2 (high expression vs. low expression) in a meta-analysis using the PrognoScan database. Cox-p denotes the *p* value obtained from the Cox repression test. (**B**,**C**) Kaplan-Meier analyses for RGL2 transcript using disease-free survival (DFS) and overall survival (OS) under a minimized p value against CRC cohorts from the GSE17537/GSE17536 datasets (**B**) and the TCGA database (**C**). (**D**) Representative immunohistochemistry (IHC) scoring for the protein intensity of RGL2 (left) and the patient number in each IHC score (right). (**E**) Kaplan-Meier analysis for the low (scores 0 and 1) and high (scores 2 and 3) protein levels of RGL2 determined by IHC experiment against the enrolled CRC patients in the commercial tissue microarray. (**F**) Cox regression test using univariate and multivariate models for RGL2 protein levels (high vs. low) under OS conditions against the enrolled CRC patients in the commercial tissue microarray. The ‘n.s.” denotes not significant.

To evaluate the clinical relevance of RGL2, we next performed a meta-analysis of RGL2 transcripts against patients with different cancer types in the PrognoScan database. The data showed that RGL2 upregulation significantly (*p* < 0.05) correlated with an increased hazard ratio in the enrolled CRC cohorts, as well as two cohorts with blood and breast cancer (Figure 2A). Kaplan-Meier analyses indicated that CRC patients with tumors harboring a higher RGL2 transcript exhibited poorer disease-free and overall survival rates in the GSE17537/GSE17536 CRC datasets (Figure 2B) and TCGA CRC database (Figure 2C), respectively. Moreover, another Kaplan-Meier analysis of RLG2 protein levels determined by IHC experiments (Figure 2D) demonstrated that higher protein expression of RGL2 indicates a poorer overall survival probability in CRC patients enrolled in a commercial tissue microarray (Figure 2E). Under the condition of overall survival probability, the data obtained from a Cox regression test using univariate and multivariate models demonstrated that RGL2 protein expression serves as an independent prognostic factor in CRC patients (Figure 2F). A similar view was also found in the Cox repression test for RGL2 mRNA levels using overall survival conditions in the TCGA CRC patients (Table S1).

### 2.3. RGL2 Upregulation Promotes the Metastatic Progression of CRC Cells

**Figure 3:**
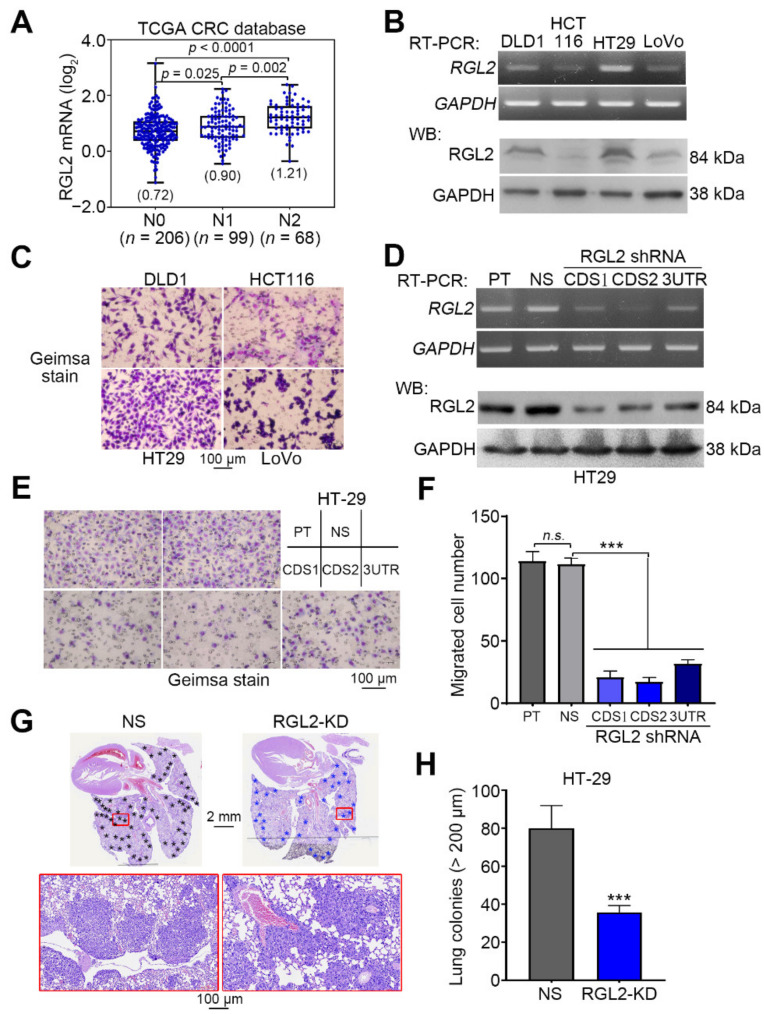
RGL2 expression is causally associated with the metastatic potential of CRC cells. (**A**) Boxplot for the transcriptional profiling of RGL2 in the TCGA CRC samples from patients with different pathologic N stages. (**B**) Reverse transcription-PCR (RT-PCR) and Western blot analyses of the mRNA and protein levels, respectively, of RGL2 and glyceraldehyde-3-phosphate dehydrogenase (GAPDH) in the selected CRC cell lines. (**C**) Geimsa staining for the migrated cells in the transwell cultivation of the selected CRC cell lines. (**D**) RT-PCR and Western blot analyses of the mRNA and protein levels, respectively, of RGL2 and GAPDH in the parental (PT), nonsilencing control (NS) and RGL2-silenced HT-29 cell variants. In (**B**,**D**) GAPDH was used as the internal control for RT-PCR and Western blot experiments. Geimsa staining (**E**) and histogram (**F**) for the migrated cells of the HT-29 cell variant shown in (**D**). Hematoxylin and eosin (H&E) staining (**G**) and histogram (**H**) of the tumor colonies (black and blue star symbols) counted from the lungs of mice transplanted with NS and RGL2-knockdown (RGL2-KD) HT-29 cells. In F and H, the “n.s.” and “***” represent not significant and *p* < 0.001, respectively. The full Western blot images of B and D are shown in Figure S1.

**Figure 4:**
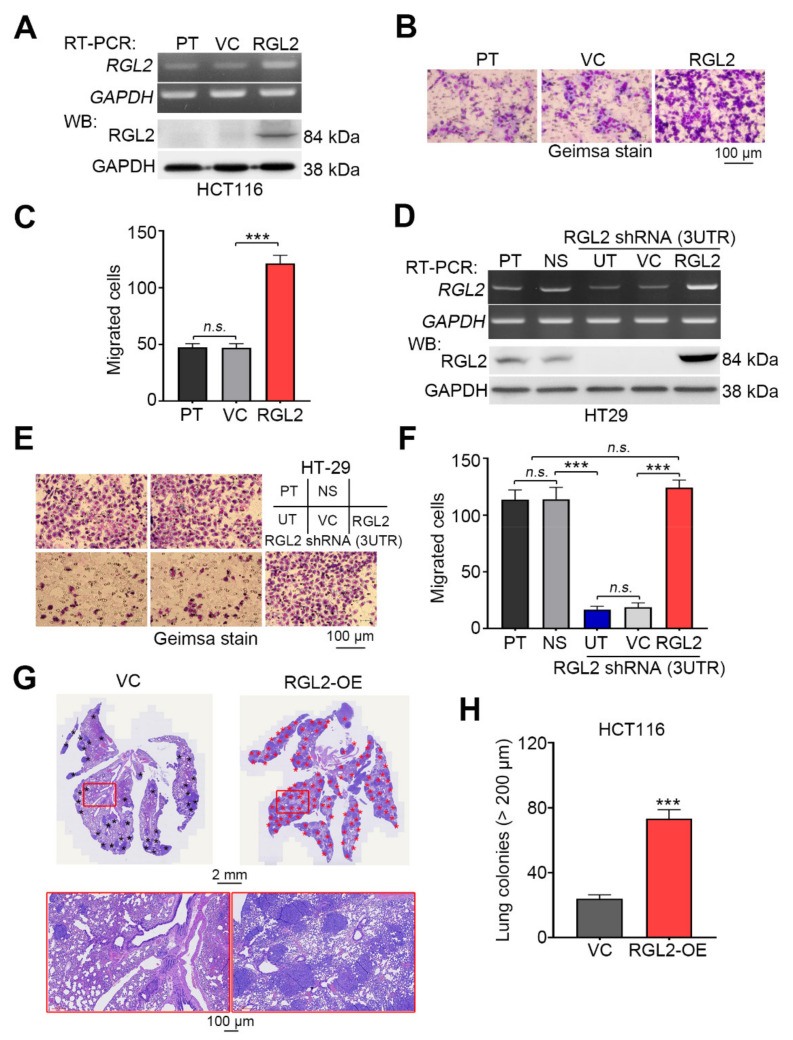
RGL2 acts as a driver in promoting the metastatic progression of CRC cells. (**A**) RT-PCR and Western blot analyses of the mRNA and protein levels, respectively, of RGL2 and GAPDH in the parental (PT), vector control (VC) and RGL2-overexpressing HCT116 cell variants. Geimsa staining (**B**) and histogram (**C**) for the migrated cells of the HCT116 cell variant shown in (**A**). (**D**) RT-PCR and Western blot analyses of the mRNA and protein levels, respectively, of RGL2 and GAPDH in PT and NS control HT-29 cells and RGL2-silenced HT-29 cells without (untreated, UT) or with transient transfection of vector control or vector-containing RGL2 gene. In (**A**,**D**), GAPDH was used as the internal control for RT-PCR and Western blot experiments. Geimsa staining (**E**) and histogram (**F**) for the migrated cells of the HT-29 cell variant shown in D. H&E staining (**G**) and histogram (**H**) of the tumor colonies (black and red star symbols) counted from the lungs of mice transplanted with VC and RGL2-overexpressing (RGL2-OE) HCT116 cells. In C, F and H, the “n.s.” and “***” denote not significant and *p* < 0.001, respectively. The full Western blot images of A and D are shown in Figure S1.

We next examined the transcriptional profiling of RGL2 in primary tumors derived from the TCGA CRC patients with different pathologic N stages. The data showed that RGL2 upregulation highly correlated with the progression of lymph node metastasis in CRC patients (Figure 3A). Similarly, we found that the endogenous mRNA and protein levels of RGL2 (Figure 3B) were causally associated with cellular migration ability (Figure 3C) in the detected CRC cell lines. The gene knockdown of RGL2 with three independent shRNA clones dramatically reduced RGL2 mRNA and protein levels (Figure 3D) and cellular migration ability (Figure 3E,F) compared to the control groups in HT-29 cells that express a relatively higher mRNA and protein levels of RGL2 (Figure 3B) and stronger migration ability (Figure 3C) in a panel of CRC cell lines. Importantly, RGL2 knockdown also significantly (*p* < 0.001) suppressed the lung colony-forming ability of HT-29 cells in the animal experiments (Figure 3G,H). Conversely, the enforced expression of the exogenous RGL2 gene predominantly elevated the intracellular mRNA and protein levels of RGL2 (Figure 4A) and enhanced the cellular migration ability (Figure 4B,C) compared with the control cell variants in HCT116 cells that harbored relatively lower RGL2 expression and exhibited a poorer migration ability in the detected CRC cells. The reconstitution of RGL2 expression by transiently transfecting an exogenous RGL2 gene into RGL2-silenced HT-29 cells showed that the repressed mRNA and protein levels of RGL2 (Figure 4D) and the suppressed cellular migration ability (Figure 4E,F) were robustly restored. In addition, RGL2 overexpression predominantly fostered the lung metastatic ability of HCT116 cells (Figure 4G,H). These findings suggest a driver function of RGL2 in promoting the metastatic progression of CRC.

### 2.4. RGL2 Induces the Activation of the Wnt/β-Catenin Pathway and EMT Progression and Thereby Promotes CRC Metastasis

**Figure 5:**
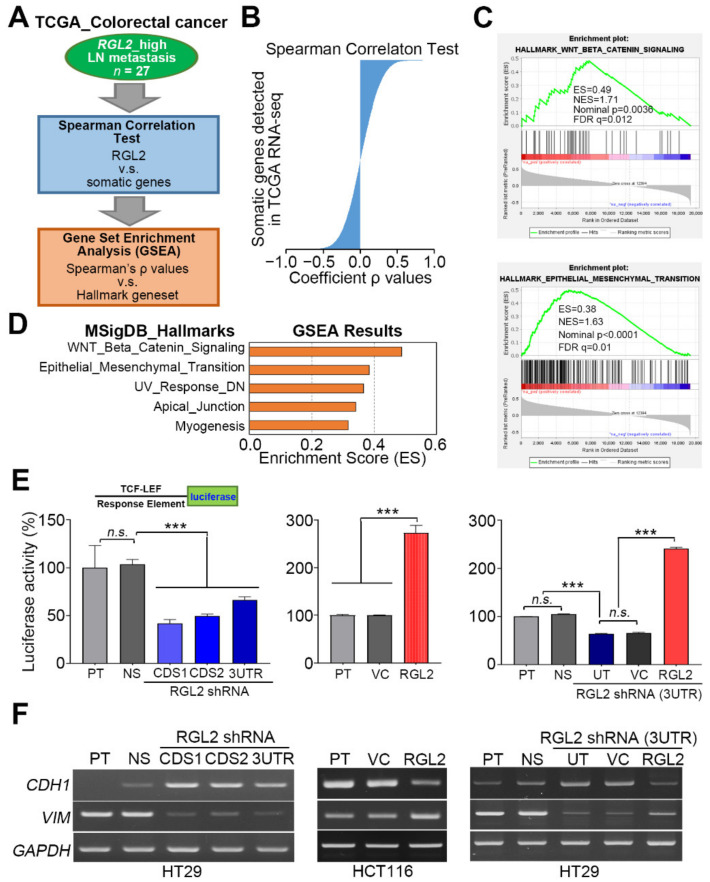
RGL2 upregulation correlates with the activation of the Wnt/β-catenin signaling axis and EMT progression in CRC. (**A**) The flowchart of generating the RGL2 signature and performing GSEA simulation. (**B**) Spearman’s coefficient ρ-values obtained from RGL2 coexpression with other somatic genes detected by RNA-sequencing experiments in the enrolled TCGA CRC samples. (**C**) The enrichment score (ES) derived from the correlation among the Wnt/β-catenin and EMT gene sets and the queried Spearman’s correlation coefficient ρ is plotted (green curve). NES and FDR denote the normalized enrichment score and the false discovery rate, respectively. (**D**) Histogram for the enrichment scores of GSEA simulation against the RGL2 signature and hallmark gene sets. (**E**) Histogram for the luciferase activity, relative to parental cells, measured in the HT-29 (left and right) and HCT116 (middle) cell variants transfected with luciferase reporter vector harboring a TCF-LEF response element for 24 h. The “n.s.” and “***” represent not significant and *p* < 0.001, respectively. (**F**) RT-PCR experiments for the CDH1 and VIM mRNA levels in the indicated HT-29 (left and right) and HCT116 (middle) cell variants. GAPDH was used as an internal control of experiment.

To ascertain the possible mechanism for RGL2-promoted CRC metastasis, we performed Spearman’s correlation test against RGL2 coexpression with other somatic genes detected by RNA-sequencing experiments in the TCGA CRC samples with higher RGL2 expression in Kaplan-Meier analysis and a record of lymph node metastasis (Figure 5A). Then, we used the ranked Spearman’s coefficient *p*-values (Figure 5B) as the RGL2 signature to perform a computer simulation using the Gene Set Enrichment Analysis (GSEA) program against the Hallmark gene set deposited in the Molecular Signature Database. GSEA results revealed that the obtained RGL2 signature highly correlated with the activation of the Wnt/β-catenin pathway and EMT progression in the enrolled TCGA CRC samples (Figure 5C,D). A luciferase-based promoter assay demonstrated that RGL2 knockdown significantly (*p* < 0.001) diminished the binding of β-catenin to the TCF-LEF response element within the upstream promoter region of the luciferase gene and thereby suppressed luciferase activity in HT-29 cells compared to control cell variants (Figure 5E, left). In contrast, RGL2 overexpression robustly elevated the luciferase activity owing to the enhanced DNA binding of β-catenin in the examined HCT116 cells compared to the control cell variants (Figure 5E, middle). The reconstitution of RGL2 expression in the RGL2-silenced HT-29 cells predominantly rescued the DNA binding of β-catenin, as shown by the strongly elevated luciferase activity in the detected HT-29 cell variant compared to the control cells (Figure 5E, right). Moreover, RT-PCR results demonstrated that RGL2 knockdown dramatically suppressed EMT progression, as judged by enhanced CDH1 (E-cadherin) levels but repressed VIM (vimentin) expression in HT-29 cells (Figure 5F, left), whereas RGL2 overexpression potentiated EMT progression, as shown by the reduced CDH1 expression but an increased level of VIM (Figure 5F, middle). Accordingly, the reconstitution of RGL2 expression in RGL2-silenced HT-29 cells reinforced EMT progression, as determined by the repressed CDH1 expression and an enhanced level of VIM (Figure 5F, right).

### 2.5. RGL2 Upregulation Enhances the Protein Stability of β-Catenin and KRAS in Metastatic CRC Cells

**Figure 6:**
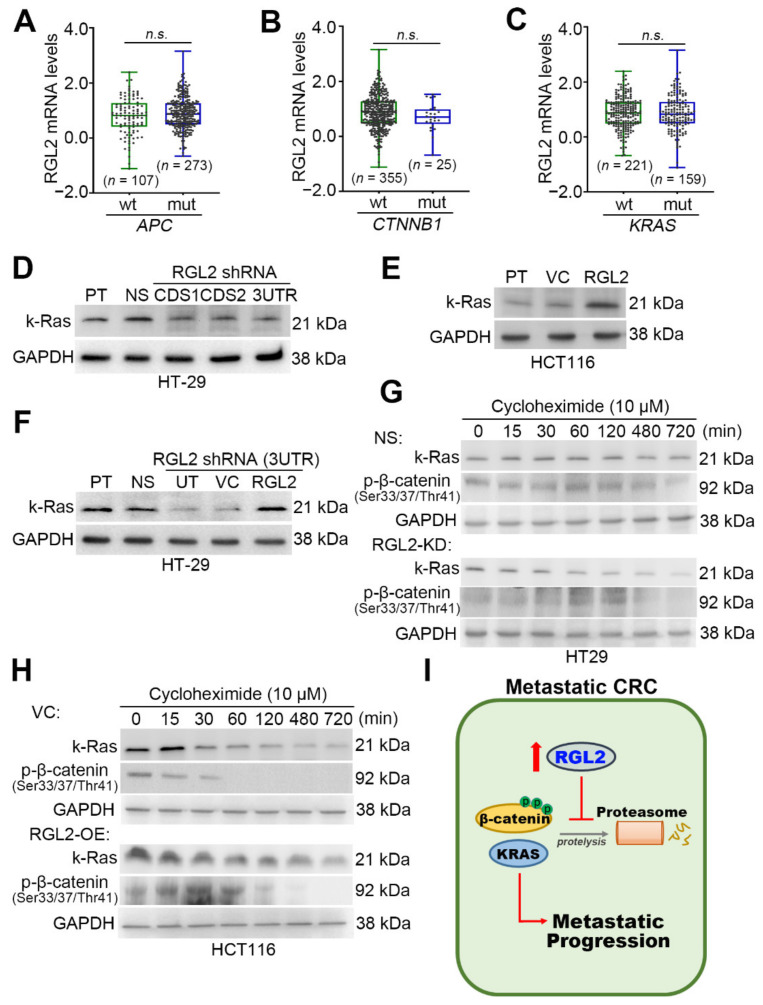
RGL2 expression is irrelevant to KRAS mutation but regulates the protein levels of KRAS in CRC. Boxplot for the transcriptional profiling of RGL2 in the TCGA samples without (wild-type, wt) or with (mutant, mut) *APC* (**A**), β-catenin (*CTNNB1*, (**B**) and KRAS (**C**) mutations. The “n.s.” denotes not significant. Western blot analyses for the protein levels of KRAS and GAPDH in the designated HT-29 (**D**,**F**) and HCT116 (**E**) cell variants. (**G**,**H**) Western blot analyses of the protein levels of KRAS, phosphorylated β-catenin at Ser33/37/Thr41 and GAPDH in cycloheximide (10 μM)-treated NS/RGL2-KD HT-29 and VC/RGL2-OE HCT116 cell variants at the indicated time intervals. In (D), (E), (F), (G), and (H), GAPDH was used as an internal control for protein loading. (**I**) An illustration of the proposed model for RGL2-promoted metastatic progression in CRC. The full Western blot images of D, E, F, G and H are shown in Figure S2.

Approximately 50% of CRC carries a KRAS mutation, while approximately 90% and 5% of CRC harbor mutations in APC and β-catenin, respectively. KRAS, APC, and β-catenin mutations in colon cancers have been associated with poorer survival and increased tumor aggressiveness [10]. We next dissected the transcriptional profiling of RGL2 in the TCGA CRC samples without or with APC, β-catenin and KRAS mutations. The data showed that RGL2 mRNA levels were not significantly different between CRC samples harboring wild-type and mutant APC, β-catenin and KRAS genes (Figure 6A–C). Intriguingly, RGL2 knockdown dramatically suppressed the protein levels of KRAS in highly metastatic HT-29 cells (Figure 6D), whereas RLG2 overexpression predominantly enhanced the protein expression of KRAS in poorly metastatic HCT116 cells (Figure 6E). Moreover, the reconstitution of RGL2 expression in RGL2-silenced HT-29 cells robustly restored the intracellular protein levels of KRAS (Figure 6F). We next pretreated HT-29 cells with or without RGL2 knockdown and HCT116 cells with or without RGL2 overexpression with cycloheximide, an inhibitor of protein synthesis, at 10 μM for the designated time periods prior to performing Western blot analyses to determine whether the RGL2-enhanced protein expression of KRAS was associated with the posttranslational modification machinery. The data showed that RGL2 knockdown accelerated the protein degradation of KRAS in the cyloheximide-treated HT-29 cell variants (Figure 6G); conversely, RGL2 overexpression extended the protein expression of KRAS in the cyloheximide-treated HCT116 cell variants (Figure 6H). Since a previous report has shown that the interaction of KRAS with β-catenin prevents the protein degradation of β-catenin [14], we further examined the protein levels of β-catenin in the presence of cyloheximide in the HT-29 and HCT116 cell variants. The data showed that RGL2 knockdown in HT-29 cells (Figure 6G) promoted but RGL2 overexpression in HCT116 cells (Figure 6H) compromised the protein degradation of β-catenin, as determined by the protein levels of phosphorylated β-catenin at Ser33/37/Thr41. The phosphorylation of Ser33/37/Thr41 residues have been shown to be critical for β-catenin degradation [15]. Based on our findings, we proposed that RGL2 upregulation promotes CRC metastasis by preventing the protein degradation of KRAS and β-catenin (Figure 6I).

## 3. Discussion

The prevalence of KRAS mutations in comparison with the prevalence of the NRAS and HRAS isoforms is relatively higher in CRC [16]. KRAS mutations have been correlated with malignant progression, e.g., drug resistance and metastasis in CRC. Here, we found that RGL2 expression is not relevant to the KRAS mutation in the TCGA CRC patients. Furthermore, RGL2 is upregulated in CRC compared to normal adjacent tissues. RGL2 upregulation correlated with a poorer prognosis in CRC patients. The current study is the first to demonstrate the clinical relevance of RGL2 in CRC. A previous study observed the upregulation of RGL2 expression in the tumor tissue and cell lines of pancreatic ductal adenocarcinoma (PDAC). Interfering RNA suppression of RGL2 reduced steady-state Ral activity, growth and invasion in PDAC cells [13]. The downstream effectors of Ras proteins, including Ras-related protein Ral-A (RalA) and Ras-related protein Ral-B (RalB), are activated by RalGEF. The RalGEF-Ral signaling pathway caused Ras-induced transformation of human cells [17]. RGL2, which is one of six RalGEFs, harbors Ras-binding domains and directly signals downstream Ras proto-oncogenes toward Ral GTPases [18]. Santos et al. [19] indicated that silencing RGL2 inhibited anchorage-dependent and independent cell growth in human non-small cell lung carcinoma (NSCLC). Moreover, the inhibition of RalGEF with PH domain and SH3 domain-binding motif 2 (RalGPS2), which is a Ras-independent RalGEF, induced apoptosis and cell cycle arrest in NSCLC cells. In addition, RalGPS2 affected several important cell cycle regulators, including the E3 ubiquitin protein ligase S-phase kinase-associated protein 2 (Skp2) and the cell cycle inhibitors p27 and p21 [19].

Recent evidence shows that inhibition of RalA expression suppresses the transformation and growth of human pancreatic cancer cells. Furthermore, RalB was required for metastasis and invasion in vitro and in vivo [20]. Active Ras signals to RalB by RGL1 and RGL2 promote invasiveness, and the contribution of RalB may be more crucial than that of the PI3K and MAPK pathways. In addition, RalB expression at the protein level in breast cancer increased in a manner consistent with progression toward metastasis [21]. Dysregulation of motility and adhesion of cells are important events in the development of metastasis. Another recent study concluded that CycD1-Cdk4 enhanced cell motility and detachment by increasing the phosphorylation of RGL2 [22]. In the present study, RGL2 upregulation promoted the metastatic progression of CRC cells. RGL2 knockdown significantly suppressed the lung colony-forming ability of HT-29 cells in animal experiments. Conversely, forced expression of the exogenous RGL2 gene enhanced the cellular migration ability of CRC cells. GSEA results revealed that the obtained RGL2 signature highly correlates with EMT progression in the TCGA CRC samples. RGL2 knockdown dramatically suppresses EMT progression by enhancing CDH1 levels but represses VIM expression in HT-29 cells. However, the mechanisms through which RGL2 enhances metastasis in CRC are unclear. Hyperactivation of Wnt/β-catenin signaling is a common feature in most CRC patients, and this pathway is an important regulator of CRC development and metastasis [23]. We found that RGL2 promoted CRC metastasis via activation of the Wnt/β-catenin pathway.

In our current study, RGL2 expression was irrelevant to KRAS mutation but regulated the protein levels of KRAS in CRC. Furthermore, the RGL2-enhanced protein expression of KRAS is associated with the posttranslational modification machinery. A previous report demonstrated that the interaction of KRAS with β-catenin inhibited the protein degradation of β-catenin [14]. RGL2 knockdown in HT-29 cells promoted the protein degradation of β-catenin. The direct interaction of β-catenin with RAS protein has been shown to promote RAS degradation by glycogen synthase kinase 3 beta (GSK3β)-mediated polyubiquitination-dependent proteasomal degradation in HCT116 cells harboring wild-type β-catenin [14]. GSK3β is a key component of the β-catenin destruction complex. Costabilization of RAS and β-catenin, particularly the KRAS mutant form, increased the growth of CRC, and high levels of RAS and β-catenin were found in CRC patient tissues [24,25]. The degradation of β-catenin and RAS provides pathological significance and a mechanical basis for the enhancement of colorectal tumorigenesis [14]. Here, we found that RGL2 expression affected the protein stability of KRAS in CRC cells. RGL2 upregulation enhances the protein stability of β-catenin and KRAS in metastatic CRC cells.

## 4. Materials and Methods

### 4.1. Clinical and Molecular Data for CRC Patients

The clinical data and overall survival (OS) time for the TCGA colorectal cancer patients were collected from the UCSC Xena website (UCSC Xena. Available online: http://xena.ucsc.edu/welcome-to-ucsc-xena/) (accessed on 1 February 2021). The molecular data obtained by RNAseq (polyA þ Illumina HiSeq, Illumina, San Diego, CA, USA) analysis of the TCGA colorectal cancer cohort were also downloaded from the UCSC Xena website. Microarray results with accession numbers GSE17536 and GSE17537 and the related clinical data were obtained from the Gene Expression Omnibus (GEO) database on the NCBI website (https://www.ncbi.nlm.nih.gov/geo/; accessed on 1 February 2021). The raw intensities in the CEL files were normalized by robust multichip analysis (RMA), and fold-change analysis was performed using GeneSpring GX11 (Agilent Technologies, Santa Clara, CA, USA). Relative mRNA expression levels were normalized by the median of all samples and presented as log_2_ values.

### 4.2. Cell Lines and Cell Culture Condition

CRC cell lines DLD1, HT-29, HCT116 and LoVo were were obtained from American Type Culture Collection (ATCC) and maintained in conditioned media supplemented with 10% fetal bovine serum (FBS) and incubated at 37 °C with 5% CO_2_. All media and supplements, e.g., FBS, were purchased from Gibco Life Technologies (Thermo Fisher Scientific Inc., Waltham, MA, USA).

### 4.3. MTT Assay

Cells (5 × 10^4^/mL) were seeded into a 96-well culture plate. After incubation, 10 μL of 3-(4,5-dimethylthiazol-2-yl)-2,5-diphenyltetrazolium bromide (MTT) (Molecular Probe, Thermo Fisher Scientific Inc., Waltham, MA, USA) stock solution was added to each well. The conversion of MTT to formazan by viable cells was performed at 37 °C for another 4 h. After the reaction, 100 μL of DMSO solution was added to each well to solubilize the formazan precipitates. The levels of formazan were determined by optical density at 540 nm using an ELISA reader (Molecular Devices, San Jose, CA, USA) to calculate cell survival rates.

### 4.4. Plasmid Construction

The gene encoding RGL2 was amplified from human cDNA (Invitrogen) using the standard polymerase chain reaction (PCR) procedure with paired primers and subcloned into the pDONR221 (Invitrogen, Thermo Fisher Scientific Inc., Waltham, MA, USA) or pLAS3w vector (National RNAi Core Facility Platform in Taiwan). The pDONR221 plasmid was recombined with the destination vector pLenti6.3/V5-DEST in 293T cells to create packaged lentiviral particles. A commercially available plasmid pLenti/GFP sharing the same backbone (Invitrogen) was used as a control. The recombinant lentiviruses in the culture medium were harvested, concentrated using a Lenti-X Concentrator (Clontech, Shiga, Japan), and then titrated by determining the viral RNA genome content with a Lenti-X qRT-PCR Titration kit (Clontech) according to the manufacturer’s manual.

### 4.5. Lentivirus-Driven shRNA Infection

Lentiviral shRNA constructs were purchased from the National RNAi Core Facility Platform in Taiwan. Lentiviruses were produced by co-transfecting the shRNA-expressing vector with the pMDG and p△8.91 constructs into 293T cells using a calcium phosphate transfection kit (Invitrogen, Thermo Fisher Scientific Inc., Waltham, MA, USA). After incubation for 48 to 72 h, media were collected as viral stocks. Cells (50% confluence) grown in 6-well plates were bathed in fresh media containing 5 μg/mL polybrene Santa Cruz, Dallas, TX, USA) before infection with a lentiviral viral particle-driven control or candidate gene shRNA at a multiplicity of infection (MOI) of 2 to 10 overnight. To select cells stably expressing the control or candidate gene shRNA, cells were further cultivated in the presence of puromycin (10 μg/mL) for 24 h. Cell lystates from the puromycin-resistant cells were subsequently subjected to Western blot analysis to confirm the efficiency of gene knockdown.

### 4.6. Reverse Transcription PCR (RT-PCR)

Total RNA was extracted from cells using a TRIzol extraction kit (Invitrogen). Aliquots (5 μg) of total RNA were treated with M-MLV reverse transcriptase (Invitrogen) and then amplified by PCR procedure using Taq-polymerase (Protech, Taipei, Taiwan) and paired primers (for RGL2, forward-GAAGAGGAGGAGGAAGAAGAAGAG and reverse-GCTACCTCTGTTGTCCTCTCTAGTTC; for CDH1, forward-GGCACAGATGGTGTGATTACAGTC and reverse-CAGGGTGAGAGAAGAGAGTGTATGT; for VIM, forward-CTGTGAAGTGGATGCCCTTAAAGG and reverse-CAAGGTCATCGTGATGCTGAGAAG; for GAPDH, forward-AGGTCGGAGTCAACGGATTTG and reverse-GTGATGGCATGGACTGTGGTC).

### 4.7. Western Blotting Analysis

Aliquots of total protein (20–100 μg) from tested cell variants were separated by SDS-PAGE and then transferred to PVDF membranes. The membranes were subsequently incubated with blocking buffer (5% nonfat milk in TBS containing 0.1% Tween-20) for 2 h at room temperature, primary antibodies against RGL2 (Genetex), phosphorylated (Ser33/37/Thr41) β-catenin/KRAS (Cell Signaling) and GAPDH (AbFrontier) overnight at 4 °C, and peroxidase-labeled species-specific secondary antibodies for 1 h at room temperature. At each step, the membranes were extensively washed. Immunoreactive bands were finally visualized by an enhanced chemiluminescence system (Amersham Bioscience, Tokyo, Japan).

### 4.8. Promoter Reporter Assay

Cells grown on 12-well plates at 70% confluence at cell density were transiently transfected with the pGL4 (Luc2PCP/TCF/LEF-RE/Hygro) vector (0.25 μg) using Lipofectamine 2000 (Invitrogen), according to the manufacturer’s protocol. The p4.74-RLuc vector (Promega, Madison, WI, USA) at 0.0125 μg was cotransfected as an internal control for transfection efficiency. After 24 h of transfection, cells were lysed in Dual-Glo^TM^ Luciferase (Promega) reagent. The firefly luciferase reaction was quenched by the addition of the Dual-GLO^TM^ Stop and Glo® Reagent (Promega). The luciferase activities were then measured by chemiluminescence in a luminometer (Packard LumiCountTM BL 10001) using the Dual-Glo® Luciferase Assay System (Promega) according to the manufacturer’s protocol.

### 4.9. Migration Assay

Polycarbonate filters were coated with human fibronectin on the lower side. Medium containing 10% FBS was added to each well of the lower compartment of the chamber. Cells were suspended in serum-free medium containing 0.1% FBS and then loaded into each well of the upper chamber. After 16 h, cells were fixed with methanol and then stained with Giemsa. Cells that migrated to the lower side of the membrane were counted under a light microscope (×200, 10 random fields in each well). All experiments were performed in quadruplicate.

### 4.10. Immunohistochemistry Staining

A paraffin-embedded tissue microarray of CRC purchased from SuperBioChips (SuperBioChips Laboratories, Seoul, Korea) in accordance with institutional review board approval (CTH-101-3-5-054) and the Declaration of Helsinki were heated and deparaffinized using xylene and rehydrated in a graded series of ethanol with a final wash in tap water. Antigen retrieval was performed using Retrieval Solution (DAKO, Woodbridge, VA, USA) in a decloaking chamber (Biocare Medical, Concord, CA, USA). Endogenous peroxidase activity was quenched by hydrogen peroxide. Sections were then incubated with anti-RGL2 antibody (Genetex, Hsin-Chu, Taiwan) at 4 °C overnight. A Vectastain ABC peroxidase system (Vector Laboratories, Burlingame, CA, USA) was used to detect the reaction products.

### 4.11. Animal Experiment

NOD/SCID mice were obtained from the National Laboratory Animal Center in Taiwan and were maintained in compliance with the institutional policy. All animal procedures were approved by the Institutional Animal Care and Use Committee at Taipei Medical University. For the in vivo lung metastatic colonization assay, 1 × 10^5^ cells in 100 μL of PBS were implanted into the mice through tail vein injection. Mice were humanely killed at the endpoint of experiments.

### 4.12. Statistical Analysis

IBM SPSS Statistics 22 software (IBM, Armonk, New York, NY, USA)) was used to analyze the statistical significance. A paired *t*-test was utilized to compare RGL2 gene expression in the cancer tissues and corresponding normal tissues. Pearson’s and nonparametric Spearman’s correlation tests were performed to estimate the association among the mRNA levels of FOXD1, G3BP2, PL-D1, and TXNIP in the detected primary tumors. Evaluation of survival probabilities was determined by Kaplan-Meier analysis and log-rank test. Student’s *t*-test, paired *t*-test, and one-way ANOVA with Tukey’s test were used to estimate the statistical significance of the detected gene expression in clinical samples. The nonparametric Friedman test was used to analyze the nonparametric data. *p* values < 0.05 in all analyses were considered statistically significant.

## 5. Conclusions

We identified that RGL2 is upregulated in primary tumors compared to normal tissues derived from CRC patients. RGL2 upregulation is related to poorer prognosis in CRC patients. Moreover, RGL2 expression was associated with the metastatic potential of CRC cells. RGL2 promoted the metastatic progression of CRC cells. In addition, RGL2 induced the activation of the Wnt/β-catenin pathway and EMT progression and thereby promoted CRC metastasis. This is the first study to document that RGL2 is capable of stabilizing KRAS and β-catenin signaling in CRC. These findings suggest that RGL2 promotes the metastasis of CRC and serves as a poor prognostic biomarker in CRC.

## Data Availability

Publicly available datasets GSE8671, GSE17536 and GSE17537 were analyzed in this study and can be found here: https://www.ncbi.nlm.nih.gov/geo/ accessed on 1 February 2021.

## Supplementary Materials

The following are available online at https://www.mdpi.com/article/10.3390/cancers13081763/s1, Table S1. Cox univariate and multivariate analyses under the condition of overall survival probability in association with RGL2 mRNA expression levels and pathological stage derived TCGA cohort with colon cancer; Figure S1. Uncut blots for Figure 3 and Figure 4; Figure S2. Uncut blots for Figure 6.

## References

[1] Ferlay J., Steliarova-Foucher E., Lortet-Tieulent J., Rosso S., Coebergh J.W., Comber H., Forman D., Bray F. (2013). Cancer incidence and mortality patterns in Europe: Estimates for 40 countries in 2012. Eur. J. Cancer.

[2] Griffin M.R., Bergstralh E.J., Coffey R.J., Beart R.W., Melton L.J. (1987). Predictors of survival after curative resection of carcinoma of the colon and rectum. Cancer.

[3] The Cancer Genome Atlas Network (2012). Comprehensive molecular characterization of human colon and rectal cancer. Nature.

[4] Guinney J., Dienstmann R., Wang X., de Reyniès A., Schlicker A., Soneson C., Marisa L., Roepman P., Nyamundanda G., Angelino P. (2015). The consensus molecular subtypes of colorectal cancer. Nat. Med..

[5] Morin P.J., Sparks A.B., Korinek V., Barker N., Clevers H., Vogelstein B., Kinzler K.W. (1997). Activation of beta-catenin-Tcf signaling in colon cancer by mutations in beta-catenin or APC. Science.

[6] Park H.S., Chun Y.J., Kim H.S., Kim J.H., Lee C.K., Beom S.H., Shin S.J., Ahn J.B. (2020). Clinical features and KRAS mutation in colorectal cancer with bone metastasis. Sci. Rep..

[7] Chu P.C., Lin P.C., Wu H.Y., Lin K.T., Wu C., Bekaii-Saab T., Lin Y.J., Lee C.T., Lee J.C., Chen C.S. (2018). Mutant KRAS promotes liver metastasis of colorectal cancer, in part, by upregulating the MEK-Sp1-DNMT1-miR-137-YB-1-IGF-IR signaling pathway. Oncogene.

[8] Sakai E., Nakayama M., Oshima H., Kouyama Y., Niida A., Fujii S., Ochiai A., Nakayama K.I., Mimori K., Suzuki Y. (2018). Combined Mutation of Apc, Kras, and Tgfbr2 Effectively Drives Metastasis of Intestinal Cancer. Cancer Res..

[9] Chen J., Rocken C., Lofton-Day C., Schulz H.U., Muller O., Kutzner N., Malfertheiner P., Ebert M.P. (2005). Molecular analysis of APC promoter methylation and protein expression in colorectal cancer metastasis. Carcinogenesis.

[10] Jeong W.J., Ro E.J., Choi K.Y. (2018). Interaction between Wnt/beta-catenin and RAS-ERK pathways and an anti-cancer strategy via degradations of beta-catenin and RAS by targeting the Wnt/beta-catenin pathway. NPJ. Precis. Oncol..

[11] Hofer F., Fields S., Schneider C., Martin G.S. (1994). Activated Ras interacts with the Ral guanine nucleotide dissociation stimulator. Proc. Natl. Acad. Sci. USA.

[12] Sun Y., Zhang Y., Ren S., Li X., Yang P., Zhu J., Lin L., Wang Z., Jia Y. (2020). Low expression of RGL4 is associated with a poor prognosis and immune infiltration in lung adenocarcinoma patients. Int. Immunopharmacol..

[13] Vigil D., Martin T.D., Williams F., Yeh J.J., Campbell S.L., Der C.J. (2010). Aberrant overexpression of the Rgl2 Ral small GTPase-specific guanine nucleotide exchange factor promotes pancreatic cancer growth through Ral-dependent and Ral-independent mechanisms. J. Biol. Chem..

[14] Lee S.K., Jeong W.J., Cho Y.H., Cha P.H., Yoon J.S., Ro E.J., Choi S., Oh J.M., Heo Y., Kim H. (2018). beta-Catenin-RAS interaction serves as a molecular switch for RAS degradation via GSK3beta. EMBO Rep..

[15] Wu G., He X. (2006). Threonine 41 in beta-catenin serves as a key phosphorylation relay residue in beta-catenin degradation. Biochemistry.

[16] Cox A.D., Fesik S.W., Kimmelman A.C., Luo J., Der C.J. (2014). Drugging the undruggable RAS: Mission possible?. Nat. Rev. Drug Discov..

[17] Hamad N.M., Elconin J.H., Karnoub A.E., Bai W., Rich J.N., Abraham R.T., Der C.J., Counter C.M. (2002). Distinct requirements for Ras oncogenesis in human versus mouse cells. Genes Dev..

[18] Ceriani M., Scandiuzzi C., Amigoni L., Tisi R., Berruti G., Martegani E. (2007). Functional analysis of RalGPS2, a murine guanine nucleotide exchange factor for RalA GTPase. Exp. Cell Res..

[19] Santos O., Parrini M.C., Camonis J. (2016). RalGPS2 Is Essential for Survival and Cell Cycle Progression of Lung Cancer Cells Independently of Its Established Substrates Ral GTPases. PLoS ONE.

[20] Lim K.H., O’Hayer K., Adam S.J., Kendall S.D., Campbell P.M., Der C.J., Counter C.M. (2006). Divergent roles for RalA and RalB in malignant growth of human pancreatic carcinoma cells. Curr. Biol..

[21] Zago G., Veith I., Singh M.K., Fuhrmann L., De B.S., Remorino A., Takaoka S., Palmeri M., Berger F., Brandon N. (2018). RalB directly triggers invasion downstream Ras by mobilizing the Wave complex. Elife.

[22] Fernandez R.M., Ruiz-Miro M., Dolcet X., Aldea M., Gari E. (2011). Cyclin D1 interacts and collaborates with Ral GTPases enhancing cell detachment and motility. Oncogene.

[23] Cheriyamundath S., Ben-Ze’ev A. (2020). Wnt/beta-Catenin Target Genes in Colon Cancer Metastasis: The Special Case of L1CAM. Cancers.

[24] Jeong W.J., Yoon J., Park J.C., Lee S.H., Lee S.H., Kaduwal S., Kim H., Yoon J.B., Choi K.Y. (2012). Ras stabilization through aberrant activation of Wnt/beta-catenin signaling promotes intestinal tumorigenesis. Sci. Signal..

[25] Moon B.S., Jeong W.J., Park J., Kim T.I., Min D.S., Choi K.Y. (2014). Role of oncogenic K-Ras in cancer stem cell activation by aberrant Wnt/beta-catenin signaling. J. Natl. Cancer Inst..

