# Supplementary material for: RGL2 Drives the Metastatic Progression of Colorectal Cancer via Preventing the Protein Degradation of β-Catenin and KRAS
Source: Cancers (Basel). 2021 Apr 7;13(8):1763. doi: 10.3390/cancers13081763 (PMC8067854; doi:10.3390/cancers13081763)
Supplement: Supplementary file 1 [file cancers-13-01763-s001.pdf]

cancers MDPI

Supplementary

RGL2 drives the metastatic progression of colorectal cancer via preventing the protein degradation of β-catenin and KRAS

Meng-Shun Sun, Lan-Ting Yuan, Chia-Hao Kuei, Hui-Yu Lin, Yen-Lin Chen, Hui-Wen Chiu* and Yuan-Feng Lin*

Table S1. Cox univariate and multivariate analyses under the condition of overall survival probability in association with RGL2 mRNA expression levels and pathological stage derived TCGA cohort with colon cancer.

		Overall survival (*n* = 184)		
**Variables**	**Crude HR (95%CI)**	***P***	**Adjusted HR (95%CI)**	***p***
Age				
< 64	1	NA	1	NA
≥ 64	1.24 (0.65-2.36)	0.522	2.10 (1.02-4.34)	0.044
Gender				
female	1	NA	1	NA
male	1.03 (0.54-1.03)	0.925	0.71 (0.35-1.46)	0.353
pT				
T1-T2	1	NA	1	NA
T3-T4	4.27 (1.03-17.8)	0.046	1.90 (0.42-8.67)	0.409
pN				
N0	1	NA	1	NA
N1-N2	2.49 (1.28-4.82)	0.007	0.49 (0.13-1.85)	0.293
pM				
M0	1	NA	1	NA
M1	4.95 (2.58-9.51)	<0.001	2.98 (1.30-6.85)	0.010
stage				
I-II	1	NA	1	NA
III-IV	3.34 (1.65-6.74)	0.001	3.31 (0.65-16.9)	0.149
RGL2 expression				
low	1	NA	1	NA
high	4.73 (1.45-15.4)	0.010	3.67 (1.08-12.4)	0.037

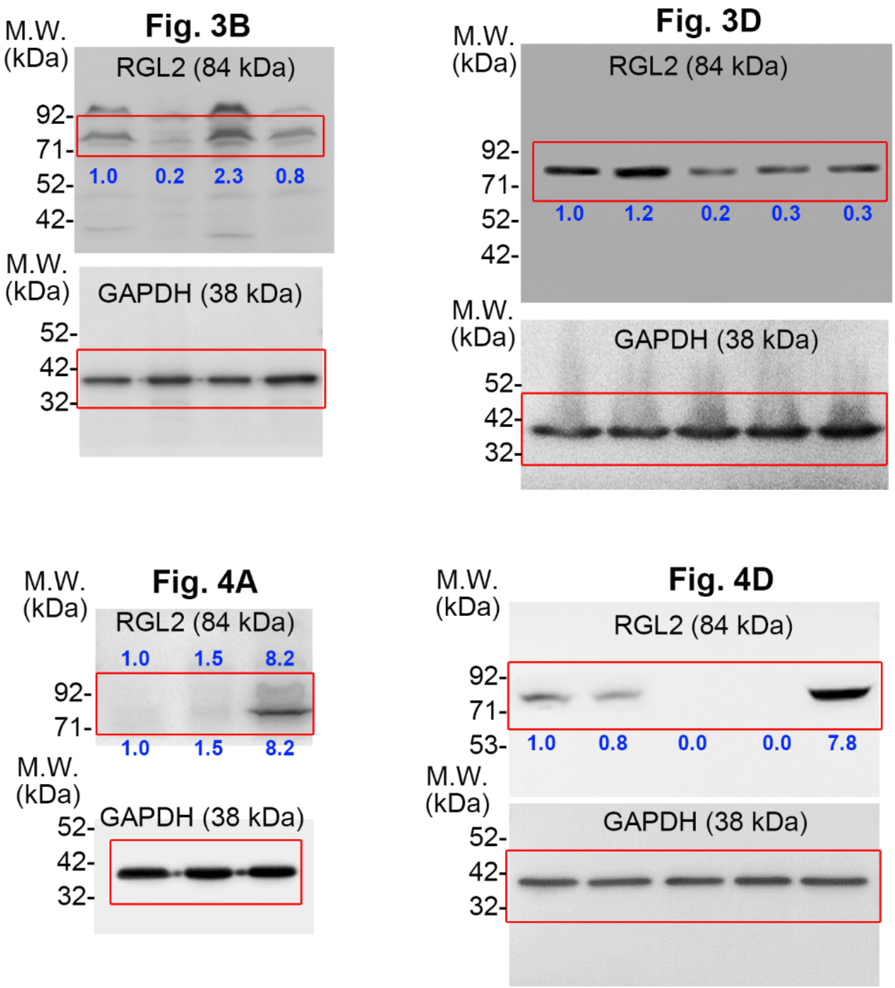

Figure S1. Uncut blots for Figure 3 and 4. The abbreviations M.W. and kDa denote molecular weight and kilodalton, respectively. The inserted values indicate the ratios of protein intensities which were normalized to the protein levels of their respective GAPDH control.

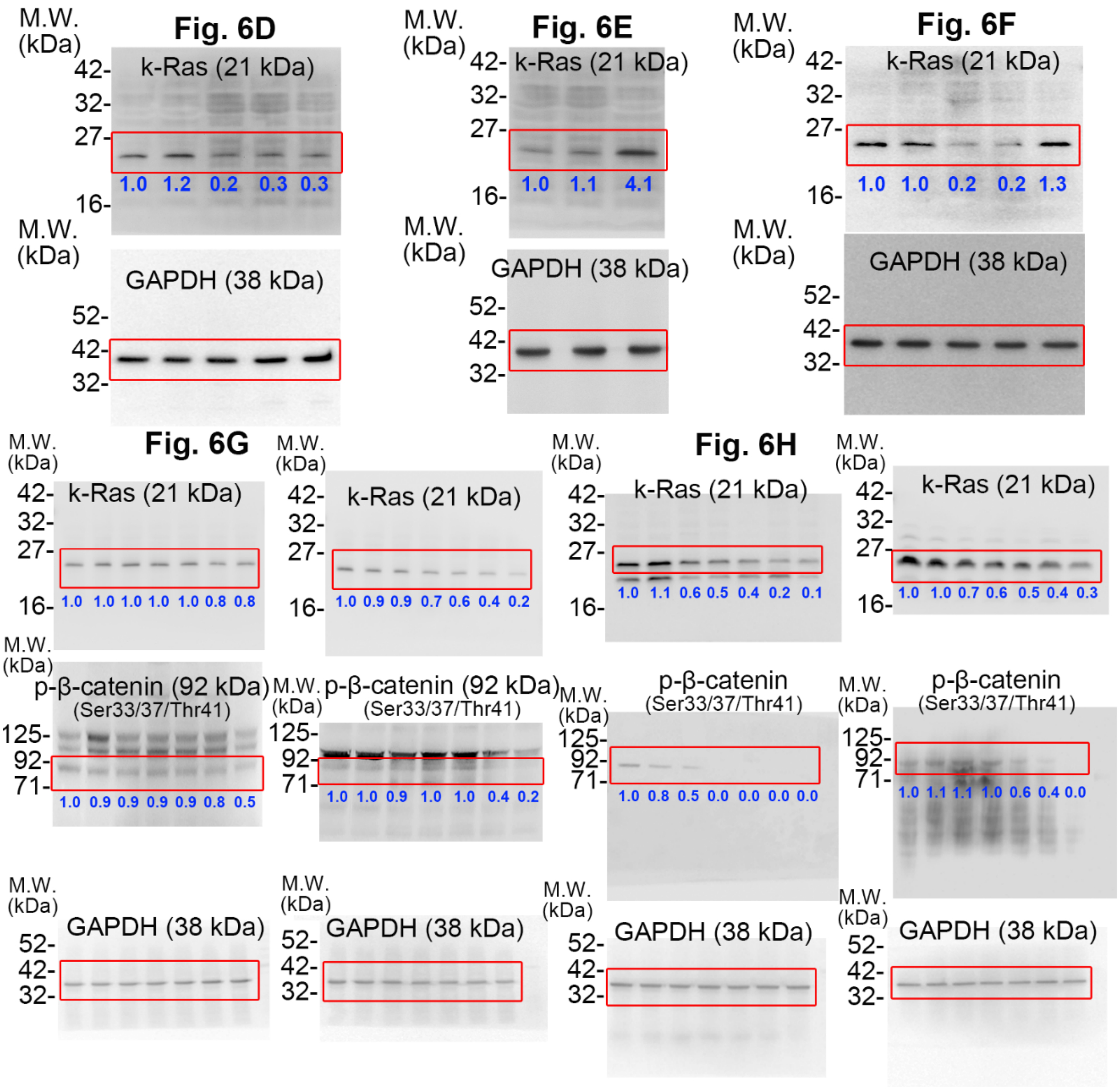

Figure S2. Uncut blots for Figure 6. The abbreviations M.W. and kDa denote molecular weight and kilodalton, respectively. The inserted values indicate the ratios of protein intensities which were normalized to the protein levels of their respective GAPDH control.
